# The Influence of Thermal Residual Stresses on Mechanical Properties of Silicon Nitride-Based Composites

**DOI:** 10.3390/ma13051092

**Published:** 2020-03-01

**Authors:** Aleksandra Dubiel, Grzegorz Grabowski, Marcin Goły, Stanisław Skrzypek

**Affiliations:** AGH University of Science and Technology, al. Mickiewicza 30, 30-059 Krakow, Poland; grzegorz.grabowski@agh.edu.pl (G.G.); marcing@agh.edu.pl (M.G.); skrzypek@agh.edu.pl (S.S.)

**Keywords:** ceramic–ceramic composites, residual thermal stresses, sin^2^ψ, FEM modeling, silicon nitride

## Abstract

In this work, two kinds of silicon nitride-based composites, namely, those with titanium nitride or silicon carbide additives, were sintered using the hot pressing technique (HP). The phase composition, microstructure, and mechanical and elastic properties of the materials were characterized. Three-dimensional geometric models of the composites were created on the basis of microstructure parameters. Using these models, bulk residual thermal stresses were calculated by the finite element method (FEM). Surface stresses were determined using the XRD method of sin^2^ψ.

## 1. Introduction

Silicon nitride-based composites are widely used structural materials. Their commercial usage is due to their great mechanical properties (high stiffness, strength, and toughness), high thermal and chemical resistance, and outstanding shock resistance [1,2]. The two most commonly used secondary phases in silicon nitride-based composites are titanium nitride and silicon carbide. Both are reported to influence the mechanical properties of composites. In Si_3_N_4_–TiN composites, an increase in fracture toughness is usually observed, but the strength of the materials may decrease [3]. There are also some works [4,5] where an increase in the strength and fracture toughness is reported. In the case of Si_3_N_4_–SiC, increased strength was observed by adding nanoparticles [6,7]. Nanoparticles located in the grain boundary phase are also reported to increase the toughness of the materials by strengthening the weak amorphous phase [8]. 

Due to the low self-diffusion rate and very strong covalent bonding, additives are often used when sintering silicon nitride [9,10]. A mixture of aluminum oxide and yttrium oxide is the most popular and effective additive. Together, they form an amorphous phase surrounding elongated silicon nitride grains. This phase strongly influences the properties of the materials [11,12,13,14]. 

When a two-phase material is sintered at high temperatures and then cooled down to room temperature, residual thermal stress may occur. The reason for such stress is the difference between the thermal expansion and elastic properties of the phases [15]. This stress affects the mechanical properties of ceramic–ceramic materials, especially fracture toughness. In silicon nitride, residual stresses have been reported to appear as a result of the mismatched thermal expansion coefficient (α) of β-Si_3_N_4_ grains and the grain boundary glassy phase, which were calculated using analytical methods by Peterson and Tien [16] and the finite element method (FEM) by Wippler and Bohlke [17]. According to their findings, residual stresses are the factors that most greatly influence the fracture toughness of polycrystalline silicon nitride. In silicon nitride-based composites, there are at least three phases with different thermal expansion coefficients in one material. This suggests that the influence of residual stresses on mechanical properties may by significant. In a study on the residual stresses in silicon nitride-based composites, Bao, Liu, and Huang [18] investigated the influence of different grain sizes, shapes, thermal expansion coefficients, and applied loads on the failure of silicon nitride-based materials. However, they did not imitate the real microstructure of silicon nitride for these simulations and they completely neglected the grain boundary glassy phase. Residual thermal stresses appear to be one of the most important factors influencing the mechanical properties of silicon nitride-based composites. Despite that, to the best of our knowledge, there are no reports of complex simulations of stresses, considering all phases in silicon nitride-based composites that have been compared with experimental mechanical testing.

In this study, two kinds of silicon nitride-based composites (Si_3_N_4_–SiC and Si_3_N_4_–TiN) were investigated. The secondary phases were chosen in such a way that, in one composite, the thermal expansion coefficient of the matrix was much smaller than the dispersed phase (α _Si3N4_ ≈ 3.5 × 10^−6^ K^−1^, α _TiN_ = 9.4 × 10^−6^ K^−1^), and in the other, the coefficient values were similar (α_SiC_ ≈ 4.4 × 10^−6^ K^−1^). Residual thermal stresses in the bulk of the materials were simulated for 3D microstructures of polycrystalline silicon nitride (reference sample) and composites containing 20 vol % of the dispersed phase. These geometric models were created based on SEM observations of real materials, considering two phases for the reference sample and all three phases present in the composites. To complete the information about stresses in composites, surface residual stresses were determined using XRD. Moreover, this complex information about stresses in materials was connected with an investigation of the microstructure and mechanical properties of silicon nitride-based composites. This research provides information about the influence of residual stresses on the mechanical properties of silicon nitride-based composites. 

## 2. Materials and Methods 

### 2.1. Preparation of Materials

Polycrystalline silicon nitride and composites with varying amounts of titanium nitride or silicon carbide, with 6 wt % aluminum oxide and 4 wt % yttrium oxide as sintering additives, were prepared from the following commercially available powders: α-Si_3_N_4_ grade M-11 higher purity (H.C. Stark, Munich, Germany), Y_2_O_3_ grade C (H.C. Stark), Al_2_O_3_ Taimicron TM-DAR (Tamei CHEMICALS), TiN grade A (H.C. Stark), and SiC UF-15 (H.C. Stark). The compositions of the prepared materials are listed in Table 1. Materials were sintered using the hot pressing (HP) technique at 1750 °C under 25 MPa pressure in nitrogen flow. Discs obtained after sintering (5 cm diameter) were cut as samples for further measurements. 

### 2.2. Materials Characterization

The density of the specimens was measured by Archimedes’s method using distilled water as the immersing medium according to ASTM C373-88. XRD measurements were taken using the PANalytical Empyrean (Malvern Panalytical, Malvern, UK) diffractometer with copper radiation (λ_Cu_ = 1.5406 Å). SEM (Nova NanoSEM 200 FEI, FEI, Tokyo, Japan) with EDS analysis was used for microstructure observation. The Young’s modulus of the materials was determined by the ultrasonic wave transition method using an ultrasonic flaw detector (Panametrics Epoch III, Olympus, Waltham, MA, USA). The hardness was determined by the Vickers indentation method using a Future FV-700 hardness tester, applying a load of 4.905 N. Fracture toughness was measured using the single-edge notched beam method (SENB), and the flexural strength was measured using a three-point bending test. The distance between supports in each measurement was 20 mm. 

### 2.3. Geometric 3D Models of Microstructure

Geometric models of the reference sample and composites containing 20 vol % of SiC or TiN were prepared based on SEM observations of microstructures. The following constituents were considered when creating the 3D models: elongated grains of silicon nitride with sizes between 0.8 and 4.9 μm and an aspect ratio (length to width) of 4, the amorphous oxide phase (consisting of Al_2_O_3_ and Y_2_O_3_) surrounding Si_3_N_4_ grains with a thickness of 0.03 μm, and the isotropic Si_3_N_4_, which refers to very small Si_3_N_4_ grains with a width below 0.2 μm. Additionally, in the composites, isometric grains (icosahedron) of the secondary phase, with sizes between 0.5 and 1.2 μm, were added. The external dimensions of the geometric model (RVE) were 5 × 5 × 5 μm. It was also assumed that grains cannot penetrate into each other.

### 2.4. Materials Data for Modeling of Residual Stresses

The elastic properties of elongated hexagonal β-Si_3_N_4_ grains and hexagonal SiC are described by six elastic constants (C_ij_), while the elastic properties of regular TiN are described by three elastic constants (C_ij_)_,_ The literature values of all C_ij_ used for modeling are listed in Table 2. For the description of thermal expansion of silicon nitride, silicon carbide, and titanium nitride in two crystallographic directions (a and c), average thermal expansion coefficients determined in the temperature range of 25–1000 °C were used. Data are presented in Table 2. 

The amorphous oxide phase formed by sintering additives, with isotropic properties, is described by Young’s modulus, the thermal expansion coefficient, and Poisson’s ratio, which are presented in Table 3. To prepare the 3D model, it was assumed that small grains of Si_3_N_4_, which are mostly isometric, also have isotropic properties. Values of Young’s modulus, Poisson’s ratio, and thermal expansion for the isotropic Si_3_N_4_ were calculated as the Hill’s average [25] based on the elastic constant (Table 2) and are presented in Table 3. 

### 2.5. Determination of Residual Stresses Using FEM

**Figure 1:**
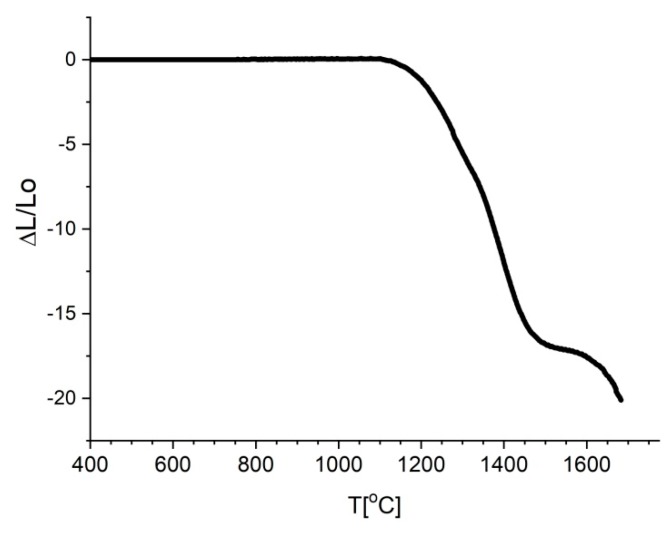
Dilatometric measurements of Si_3_N_4_ + 6%Al_2_O_3_ + 4%Y_2_O_3_.

The loads of the model were cooled from 1000 to 25 °C. The upper temperature was the temperature in which our system was stiff and created stresses that could not be relaxed. In this study, the upper temperature was selected based on dilatometric measurements of samples (Figure 1). 

The shrinkage started around 1100 °C. The temperature considered in modeling was 1000 °C due to pressure applied during the HP process. When the pressure was applied, diffusion started at lower temperatures.

Because the geometric models were periodic, periodic boundary conditions (PBCs) were used in finite element analyses. PBCs require joining displacements of corresponding nodes (being on opposite, periodic faces of the model) [27,28]. The implementation of PBCs was based on dummy nodes that matched the displacement of the corresponding model walls. The procedure used was described in detail by Grabowski [29]. The number of independent realizations, with the assumed RVE sizes, was calculated based on the differences between the mean strains recorded for perpendicular directions of the model. These differences, which were connected with the anisotropy of the material, allowed us to determine the dispersion, on the basis of which the number of necessary independent realizations was estimated to obtain results with a maximum permissible error of 1% and a confidence level of 0.95 [29]. For Si_3_N_4_, the number of realizations was five; for composite materials, due to the replacement of elongated anisotropic Si_3_N_4_ grains by isometric grains of the secondary phase, the number of realizations decreased. In the Si_3_N_4_–TiN system, in which differences in thermal expansion between phases were the largest, it was three, and for Si_3_N_4_–SiC, the number was smaller. However, it was assumed for both composites that analyses would be made for three realizations.

### 2.6. Measurement of Stresses Using the Sin^2^ψ Method

Before measurement of stresses by X-ray diffraction, samples were annealed to release stresses that were created during cutting and polishing [15]. To avoid grain growth during annealing, the applied temperature was below the Tammann temperature to only allow diffusion on grain boundaries. Samples were annealed for 2 h at 1200 °C.

The XRD measurements for stress analysis were taken using a Bruker D8 Advance diffractometer with the cobalt radiation wavelength (λ_Co_ = 1.7902 Å). The {321} diffraction line was used for determination of residual stress in Si_3_N_4_, line {400} for TiN, and {202} for SiC, and the tilt angle ψ was in the range of 0°–40°. The X-ray diffraction methods for determining residual stresses were based on the literature and our own elaborations. The average lattice strain in the *L_3_* direction was equal to [30,31,32]

(1) $$\begin{array}{l} <\varepsilon^{\prime }(\varphi ,\psi ){>}_{\left(hkl\right)}=s_{1}(hkl)(\sigma_{11}^{I}+\sigma_{22}^{I}+\sigma_{33}^{I})+\frac{1}{2}s_{2}(hkl)(\sigma_{11}^{I}\cos^{2}\varphi +\sigma_{22}^{I}\sin^{2}\varphi +\sigma_{12}^{I}\sin2\varphi )\sin^{2}\psi \\ +\frac{1}{2}s_{2}(hkl)\sigma_{33}^{I}\cos^{2}\psi +\frac{1}{2}s_{2}(hkl)(\sigma_{13}^{I}\cos\varphi +\sigma_{23}^{I}\sin\varphi )\sin2\psi \end{array}$$

where *s_1_(hkl)* and *s_2_(hkl)* are diffraction elastic constants for quasi-isotropic polycrystalline, and *σ^I^_ij_* macrostresses are defined with respect to the sample system. The general relation between the ε’(φ,ψ) and σ’*_ij_* components is usually expressed as

(2) $$\varepsilon \prime_{\varphi ,\psi }=F_{ij,\;\left(hkl\right)\varphi ,\psi }\cdot \sigma_{ij}^{I}$$

where *F_ij_* is the diffraction elastic constant for {hkl} reflection.

If the shear components of the stress tensor are neglected and the assumptions of the quasi-isotropic behavior of *F_ij_* are neglected, the simplification of the above equation for the plain stress field (assumed for the surface) can be expressed as

(3) $$\varepsilon \prime_{ij}=s_{1}\left({\sigma^{\prime }}_{11}+{\sigma^{\prime }}_{22}\right)+\frac{1}{2}s_{2}\left({\sigma^{\prime }}_{11}\cos^{2}\varphi +{\sigma^{\prime }}_{22}\sin^{2}\varphi +{\sigma^{\prime }}_{12}\sin2\varphi \right)\sin^{2}\psi$$

This simplified calculation was used to measure thermal-origin residual macrostresses in sintered samples.

## 3. Results and Discussion 

### 3.1. Materials Characterization

**Figure 2:**
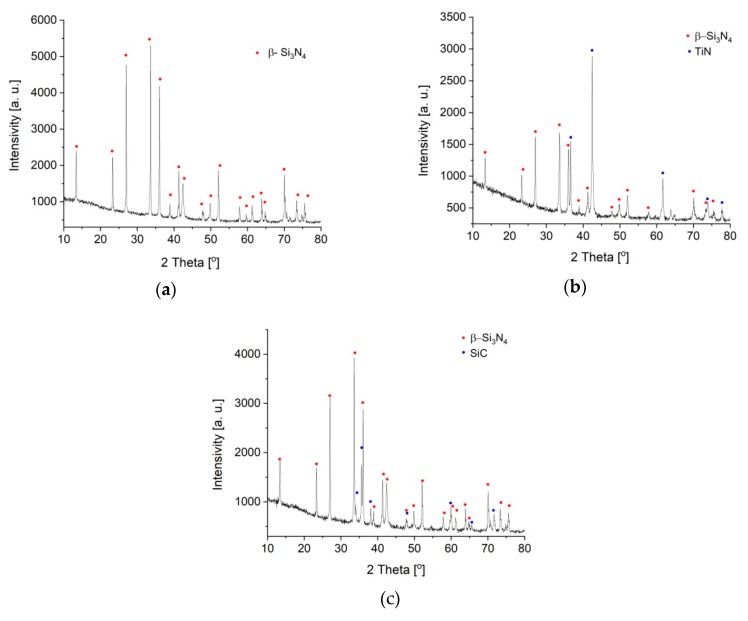
XRD patterns: (**a**) Si_3_N_4_, (**b**) Si_3_N_4_ + 20 vol % SiC, and (**c**) Si_3_N_4_ + 20 vol % TiN.

The prepared materials had high densities (Table 4). XRD analysis confirmed the presence of the assumed phases in the materials: β-Si_3_N_4_ in polycrystalline silicon nitride, and β-Si_3_N_4_ and silicon carbide or titanium nitride in the composites (Figure 2a–c). Sintering additives formed the amorphous phase, which was not visible in XRD patterns.

**Figure 3:**
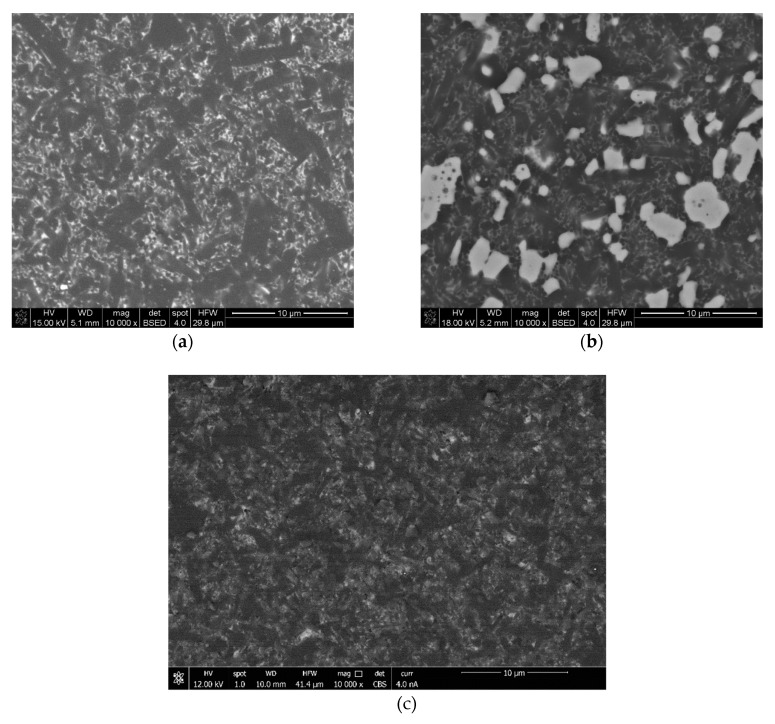
SEM observation of materials: (**a**) polycrystalline Si_3_N_4_, (**b**) Si_3_N_4_ + 20%TiN, and (**c**) Si_3_N_4_ + 20%SiC.

The SEM investigation revealed dense microstructures in all materials. Examples of images of the microstructure are shown in Figure 3. Elongated dark-grey silicon nitride grains with an aspect ratio of around 4 were surrounded by a thin layer of the white amorphous phase formed by sintering additives. There was also some amount of small isometric grains of silicon nitride visible in the microstructure. In Si_3_N_4_*–TiN systems (*Figure 3b), white TiN grains had an average size of 1.4 µm. These grains were uniformly distributed in the silicon nitride matrix. The microstructure of the matrix was similar to that of polycrystalline silicon nitride. 

In composites with SiC, the contrast between grains observed using a backscattered electron detector (BSE) was much smaller than in the case of composites with titanium nitride due to the similar chemical composition of SiC and Si_3_N_4_ and the small difference between the atomic mass of carbon and nitrogen. However, the lighter-grey grains visible in Figure 3c were silicon carbide; they were uniformly distributed in the microstructure and their average grain size was also around 1.4 µm. The microstructure of the silicon nitride matrix was also very similar to the reference sample. 

The Young’s modulus of polycrystalline silicon nitride was almost 300 GPa, and it increased with the addition of secondary phases in both types of composites, exceeding 320 GPa for the Si_3_N_4_ + 20% TiN composite. The hardness of the reference sample was 13.3 GPa. The titanium nitride additive did not cause a great increase in hardness; for the composite with 20% TiN, it was 13.6 GPa. Much greater hardness was measured for composites with silicon carbide. The hardness of Si_3_N_4_ + 20%SiC was 15.3 GPa. 

**Figure 4:**
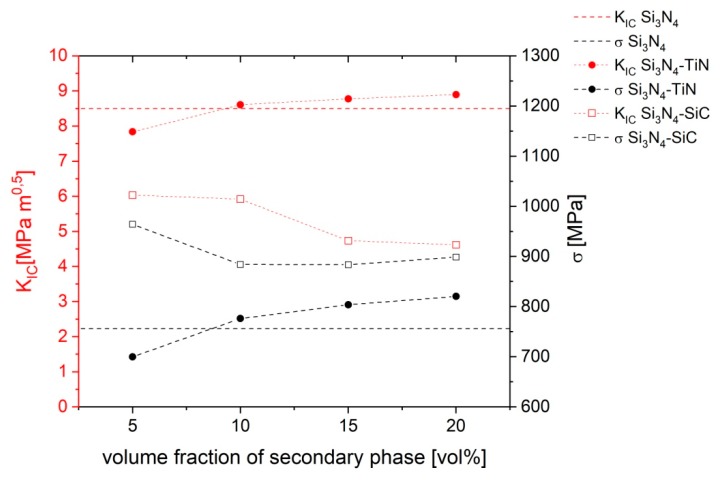
Fracture and strength of materials.

The fracture toughness of the reference sample was high (8.5 MPa∙m^0.5^). In Si_3_N_4_–TiN systems, we observed a slight increase of fracture toughness as the amount of titanium nitride increased (Figure 4). The strength of these composites also increased with higher amounts of titanium nitride (Figure 4). In Si_3_N_4_–SiC composites, the fracture toughness strongly decreased as the amount of SiC increased, while the strength increased, and for all composites, it exceeded 880 MPa (Figure 4).

### 3.2. Geometric 3D Models of Microstructure

**Figure 5:**
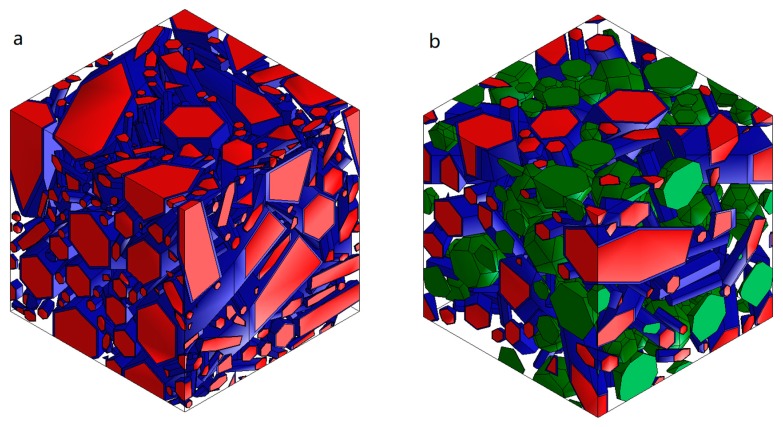
3D microstructure of (**a**) polycrystalline silicon nitride and (**b**) silicon nitride-based composite materials with 20 vol % of secondary phase; red colour- silicon nitride grains, green colour- secondary phase grains, navy blue color- amorphous oxide phase.

Models of polycrystalline silicon nitride and the composite are presented in Figure 5a,b, respectively. 

The amount of elongated silicon nitride grains in the prepared model of polycrystalline Si_3_N_4_ was 40%. It would have been possible to increase the volume fraction of these grains by reducing the size of the grains below 0.8 μm, but this would have led to a significant increase in the numerous mesh elements required for model discretization. Moreover, grains with a length below 0.8 μm have a width below 0.2 μm, so small grains are on the resolution level of SEM. In the works of Wippler [17,33], a greater number of elongated grains were created, but the author assumed that the grains can overlap and deform. The SEM observations of our research did not reveal deformed or overlapping grains, and because of that, these mechanisms were not allowed in the generation of our model. Elongated grains of silicon nitride were covered by the grain boundary amorphous phase. The volume fraction of this oxide phase in the model was 9.5%, which is in good agreement with the experiment, where this volume fraction was around 8% (10 wt % of sintering additives). The rest of the space in the model of polycrystalline silicon nitride was fulfilled by the isotropic Si_3_N_4_, representing very small silicon nitride grains visible in the microstructure. 

Models of composite materials (Figure 5b) contained an additional 20% of the secondary phase. This caused a decrease in the amount of elongated grains to 21% and, hence, a decrease in the amount of the grain boundary phase to 5%. 

### 3.3. Residual Stresses Determined Using FEM

Based on the average stresses determined using FEM, grains of Si_3_N_4_ in polycrystalline silicon nitride were under slight compression (approx. −30 MPa), while tensile stresses (approx. 270 MPa) were present in the grain boundary phase (Table 5). This behavior corresponds with the results achieved analytically by Peterson and Tien [16]. However, the values of the stresses simulated in this study were lower than those in the abovementioned article [16].

**Figure 6:**
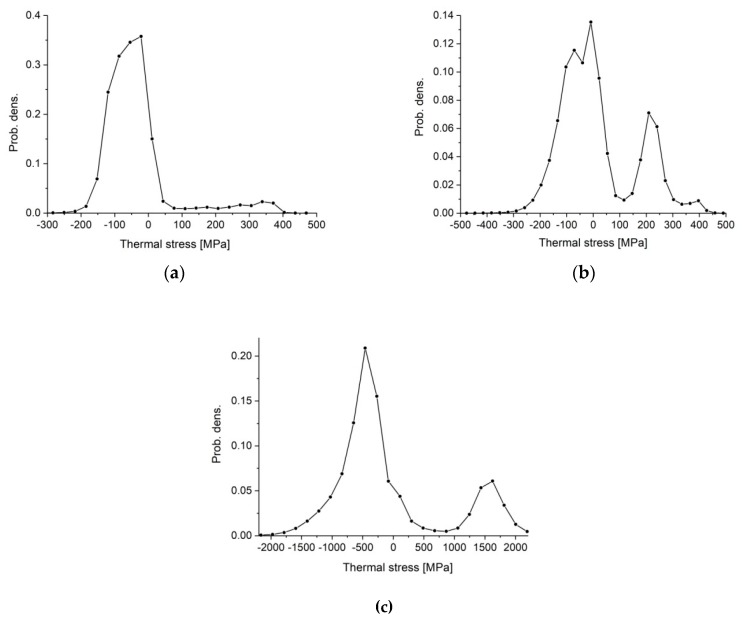
Histograms of residual stresses determined by FEM simulation for (**a**) Si_3_N_4_, (**b**) Si_3_N_4_ + SiC, and (**c**) Si_3_N_4_ + TiN.

**Figure 7:**
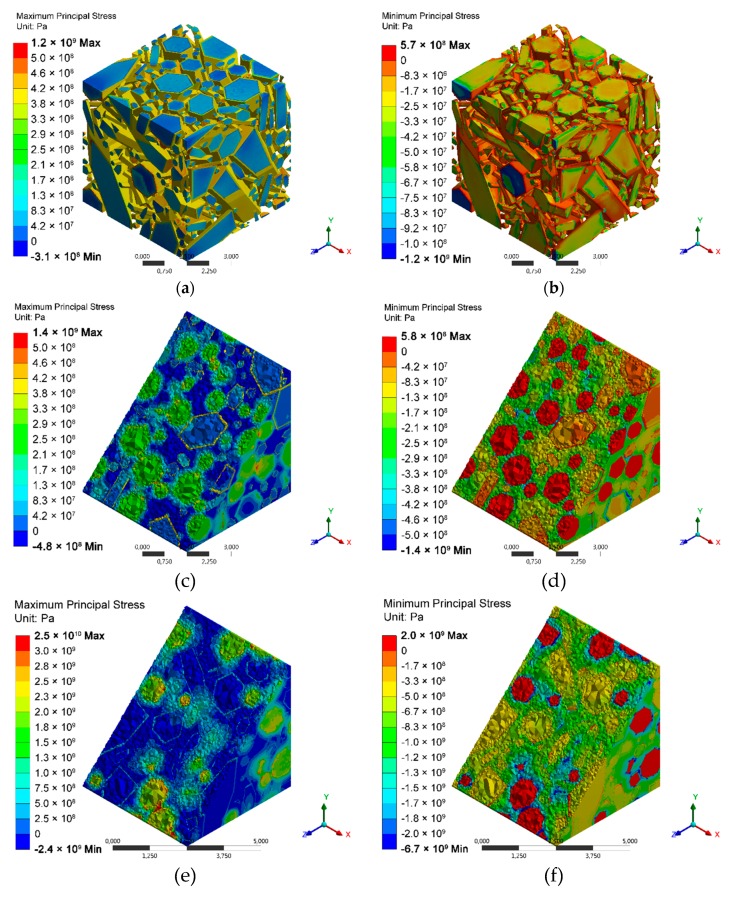
Distribution of residual thermal stresses: (**a**) maximum principal stresses in Si_3_N_4_, (**b**) minimum principal stresses in Si_3_N_4_, (**c**) maximum principal stresses in Si_3_N_4_ + 20%SiC, (**d**) minimum principal stresses in Si_3_N_4_ + 20%SiC, (**e**) maximum principal stresses in Si_3_N_4_ + 20%TiN, and (**f**) minimum principal stresses in Si_3_N_4_ + 20%TiN. Color scales and stress ranges for subsequent images are different.

According to the histogram of stresses in polycrystalline silicon nitride (Figure 6a), we can assume that, even though the values of the average stresses in the grains were around −30 MPa, these stresses were locally lower than −200 MPa. In the distribution of compressive stresses shown in Figure 7b, we can see that these minimum stress values (most compressive stresses) were located on some edges of elongated grains. Maximum tensile stresses were observed in the grain boundary phase (Figure 7a), and according to the histogram shown in Figure 6a, these stresses may locally exceed 400 MPa. 

In Si_3_N_4_–SiC composites, the average value of tensile stresses in the amorphous phase was very similar to the reference sample (Table 6). The simulation indicated that the incorporation of silicon carbide caused an increase in average compressive stresses in silicon nitride grains. Even though the average stresses were about −70 MPa, they locally exceeded -300 MPa (Figure 6b). The secondary phase was under tensile stresses with an average value of 200 MPa. According to the distribution of stresses, the highest tension (Figure 7c) was localized in the grain boundary phase, while the highest compression (Figure 7d) can be observed locally in Si_3_N_4_ between SiC grains.

The highest values of stresses were observed in Si_3_N_4_–TiN composites. The calculated average compression in silicon nitride was −400 MPa, and locally, as shown by the histogram in Figure 6c, these stresses reached −2000 MPa. Average tensile stresses in the grain boundary phase were reduced to 70.8 MPa. Titanium nitride grains were under strong tension; the calculated average stresses were around 1650 MPa, but according to the histogram, they locally exceeded 2000 MPa. The highest stresses were created on the contact of titanium nitride grains (Figure 7e). The lowest stresses were located in silicon nitride between titanium nitride grains (Figure 7f).

### 3.4. Measurement of Stresses Using the Sin^2^ψ Method

Measurements were made for polycrystalline silicon nitride and composites with 20% titanium nitride or silicon carbide with 6 wt % aluminum oxide and 4 wt % yttrium oxide. The results for Si_3_N_4_ and the secondary phases in the analyzed composites are presented in Table 6. The applied methodology enabled measurement of macrostresses for the depth of penetration of the surface layer in the range of −70 μm in the case of symmetrical Bragg–Brentano diffraction and 35–45 μm for the applied sin^2^ψ method. 

**Figure 8:**
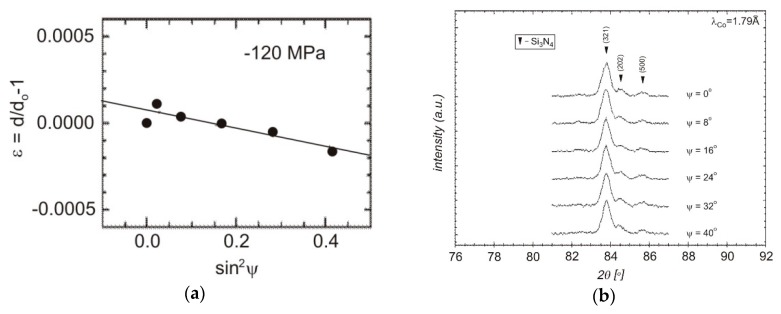
Linear relationship ε_φ__ψ_ = f(sin^2^ψ) + b in the classical sin^2^ψ method (**a**) and view of the registered line {321} for individual inclination angle ψ (**b**).

The residual stresses determined for different samples varied considerably (Table 6 and Figure 8). In polycrystalline silicon nitride, the value of normal stresses was about −160 MPa. In Si_3_N_4_–SiC composites, normal compressive stresses in silicon nitride grains were higher than in the reference sample (σ = −330 MPa), and in SiC grains, tensile stresses of about 200 MPa were measured. These results are in agreement with the stresses simulated for bulk materials. Higher values of stresses were measured for composite materials, compressive stresses were determined for Si_3_N_4_ grains, and tensile stresses were measured for silicon carbide. For these materials, values measured on the surface were higher than the values obtained by simulation for the bulk of the materials. This increase may have been caused by additional stresses introduced by material cutting and polishing. Lower values obtained in FEA simulations could also result from the assumptions made in the model: I) linear-elastic material properties, II) average values of thermal expansion coefficients in the considered temperature range. Despite the samples being relaxed, some stresses may have been induced. In Si_3_N_4_–TiN composites, only compressive stresses were measured by XRD. In comparison with stresses determined for the bulk of this composite, the values were much lower, and in the case of titanium nitride grains, tensile stresses were calculated, while compressive stresses were measured using XRD. Probably during cutting, when additional stresses were induced, the yield limit of the material was locally suppressed, which caused cracking of the overloaded zones. After such cracking, stresses were released and only some compression was measured on the surface. This effect was observed only in Si_3_N_4_–TiN composites, in which thermal residual stresses were extremely high due to the great difference in the thermal expansion coefficients. 

### 3.5. The Influence of Stresses on Mechanical Properties

The stress system in polycrystalline silicon nitride had a positive influence on the mechanical properties. The increase of fracture toughness of composites is caused by compressive stresses in grains and tensile stresses in the amorphous phase, which promotes cracking through the grain boundaries. Compressive stresses measured on the surface contribute to the strength of the materials. In composites with titanium nitride, the compressive stresses in the matrix were extremely high, which can cause annihilation of crack propagation, and owing to that, an increase of fracture. High values of stresses may also create some microcracks in the material, which beneficially influence fracture toughness. Surface stresses in this composite were also advantageous, and only compressive stresses were observed on the surface. In composites with silicon carbide, there were high compressive stresses in the silicon nitride grains and tensile stresses in the silicon carbide grains. This system of stresses caused a decrease of fracture. Probably, cracks went through tensile stress in silicon carbide grains, and low value of compression in silicon nitride was the reason of some intergranular cracking. Beneficial stress systems on the surface of these composites, and high values of compressive stresses may relax external tension, and thanks to that, cause the increase of strength. 

## 4. Conclusions

1. Simplification during preparation of the 3D geometric models, regarding replacement of the smallest Si_3_N_4_ grains by a continuous and isotropic constituent, allowed for effective calculations using the finite element method, without an excessive increase in the complexity of the calculation model (mesh density).
2. The results of the FEA simulation indicate a complex system of stress in materials based on Si_3_N_4_. The silicon nitride phase is under compressive stress and the grains of secondary phases are under tensile stress. Very high stress values for Si_3_N_4_–TiN indicate the possibility of microcracks in the material, which occur mostly on the surface and cause an increase of fracture toughness.
3. The results of XRD measurements of surface stresses confirmed the presence of compressive stress in silicon nitride grains. In the Si3N4–SiC composites, the values coincided with those obtained numerically for the solid material. In Si3N4–TiN composites, lower stress values confirmed relaxation through cracking.
4. The results of mechanical testing and stress analysis show that the high fracture toughness and strength of polycrystalline silicon nitride may be the effect of the advantageous stress system in this material. Compressive stress in grains and tensile stress in grain boundaries result in cracking through grain boundaries. Moreover, compressive stress on the surface are beneficial for the increase of the strength of materials. The investigation of Si3N4–TiN composites indicates that the system of stresses obtained by the distribution of the secondary phase with a much higher thermal expansion coefficient than the matrix has a beneficial influence on the fracture of materials. In composites with a small difference of thermal expansion coefficients between phases, the stresses were smaller and a decrease of fracture toughness was observed. On the other hand, in this kind of composite, we observed an increase of strength.

## Figures and Tables

**Table 1:** Composition of prepared materials.

Material	Si_3_N_4_ matrix [vol %] 90 wt % Si_3_N_4_, 6 wt % Al_2_O_3_, 4 wt % Y_2_O_3_	Secondary phas [vol %] (TiN/SiC)
Si_3_N_4_	100 vol %	--
Si_3_N_4_ + 5%TiN	95 vol %	5 vol %
Si_3_N_4_ + 10%TiN	90 vol %	10 vol %
Si_3_N_4_ + 15%TiN	85 vol %	15 vol %
Si_3_N_4_ + 20%TiN	80 vol %	20 vol %
Si_3_N_4_ + 5%SiC	95 vol %	5 vol %
Si_3_N_4_ + 10%SiC	90 vol %	10 vol %
Si_3_N_4_ + 15%SiC	85 vol %	15 vol %
Si_3_N_4_ + 20%SiC	80 vol %	20 vol %

**Table 2:** Elastic constant C_ij_ and thermal expansion coefficients.

Phase		C11 (GPa)	C12 (GPa)	C13 (GPa)	C33 (GPa)	C44 (GPa)	C66 (GPa)	α_a_ (K^−1^)	α_c_ (K^−1^)
β-Si_3_N_4_	[19,20]	433	195	127	574	108	119	3.23 × 10^−6^	3.72 × 10^−6^
SiC	[21,22]	478	98	56	522	148	191	4.51 × 10^−6^	4.19 × 10^−6^
TiN	[23,24]	625	165	-	-	163	-	9.35 × 10^−6^	9.35 × 10^−6^

**Table 3:** Properties of isotropic phases.

Phase	E (GPa)	υ	α_iso_ (10^−6^ K^−1^)
Isotropic Si_3_N_4_	416	0.37	3.39
Amorphous oxide phase [26]	133	0.29	5.67

**Table 4:** Density and elastic and mechanical properties of polycrystalline silicon nitride and prepared composites.

Material	Density	E (GPa)	K_IC_ (MPa∙m^0.5^)	σ (MPa)	HV (GPa)
Si_3_N_4_	3.20 ± 0.01	298 ± 4	8.5 ± 0.9	756 ± 96	13.3 ± 0.3
Si_3_N_4_ + 5%SiC	3.19 ± 0.01	302 ± 5	6.0 ± 0.8	964 ± 138	14.3 ± 0.4
Si_3_N_4_ + 10%SiC	3.19 ± 0.01	301 ± 5	5.9 ± 0.6	884 ± 203	14.4 ± 0.3
Si_3_N_4_ + 15%SiC	3.20 ± 0.01	313 ± 5	4.7 ± 0.4	884 ± 177	15.3 ± 0.5
Si_3_N_4_ + 20%SiC	3.19 ± 0.01	317 ± 5	4.6 ± 0.6	898 ± 192	15.3 ± 0.3
Si_3_N_4_ + 5%TiN	3.33 ± 0.01	298 ± 4	7.8 ± 0.4	700 ± 49	13.5 ± 0.6
Si_3_N_4_ + 10%TiN	3.40 ± 0.01	309 ± 4	8.6 ± 0.6	776 ± 46	13.5 ± 0.4
Si_3_N_4_ + 15%TiN	3.49 ± 0.01	314 ± 4	8.8 ± 0.6	803 ± 36	13.9 ± 0.4
Si_3_N_4_ + 20%TiN	3.65 ± 0.01	322 ± 12	8.9 ± 0.3	820 ± 49	13.6 ± 0.4

**Table 5:** Average residual stresses in silicon nitride-based materials: finite element method (FEM) simulation.

Material	Si_3_N_4_ [MPa]	Grain Boundary Phase [MPa]	Secondary Phase [MPa]
Si_3_N_4_	−26.6	271.0	-
Si_3_N_4_ + 20%SiC	−69.9	257.2	204.1
Si_3_N_4_ + 20%TiN	−439.1	70.8	1654.6

**Table 6:** Measured surface residual stresses in silicon nitride-based materials and secondary phases. Diffraction constant 1/2S2 (MPa^−1^) for particular phase 4.2 × 10^−6^ for Si_3_N_4_, 2.4 × 10^−6^ for SiC, and 2.8 × 10^−6^ for TiN.

Material	Base Material Si_3_N_4_		Secondary Phase	
	Normal Stress σ (MPa)	Shear Stress τ (MPa)	Normal Stress σ (MPa)	Shear Stress τ (MPa)
Si_3_N_4_	−163 ± 11	55 ± 10	---	---
Si_3_N_4_ + 20%SiC	−331 ± 35	89 ± 8	254 ± 25	−120 ± 10
Si_3_N_4_ + 20%TiN	−195 ± 12	70 ± 5	−171 ± 30	−97 ± 14
